# UnoViS: the MedIT public unobtrusive vital signs database

**DOI:** 10.1186/s13755-015-0010-1

**Published:** 2015-06-02

**Authors:** Tobias Wartzek, Michael Czaplik, Christoph Hoog Antink, Benjamin Eilebrecht, Rafael Walocha, Steffen Leonhardt

**Affiliations:** Chair of Medical Information Technology, RWTH Aachen University, Pauwelsstr. 20, 52074 Aachen, Germany; RWTH Aachen University Hospital, Pauwelsstr. 30, 52074 Aachen, Germany

**Keywords:** Capacitive ECG, cECG, Unobtrusive, Toolbox, Matlab, PhysioNet, Non-contact, Ubiquitous, Fusion

## Abstract

While PhysioNet is a large database for standard clinical vital signs measurements, such a database does not exist for unobtrusively measured signals. This inhibits progress in the vital area of signal processing for unobtrusive medical monitoring as not everybody owns the specific measurement systems to acquire signals. Furthermore, if no common database exists, a comparison between different signal processing approaches is not possible. This gap will be closed by our UnoViS database. It contains different recordings in various scenarios ranging from a clinical study to measurements obtained while driving a car. Currently, 145 records with a total of 16.2 h of measurement data is available, which are provided as MATLAB files or in the PhysioNet WFDB file format. In its initial state, only (multichannel) capacitive ECG and unobtrusive PPG signals are, together with a reference ECG, included. All ECG signals contain annotations by a peak detector and by a medical expert. A dataset from a clinical study contains further clinical annotations. Additionally, supplementary functions are provided, which simplify the usage of the database and thus the development and evaluation of new algorithms. The development of urgently needed methods for very robust parameter extraction or robust signal fusion in view of frequent severe motion artifacts in unobtrusive monitoring is now possible with the database.

## Background

Unobtrusive vital signs monitoring for daily life as well as clinical scenarios like the general ward is gaining increasing attention in research. Moreover, its market is growing, with companies starting to sell, for example, devices for personal fitness tracking. The sensor systems for unobtrusive vital signs monitoring use different physical principles to measure information about a patient [1] without the need for medical trained staff or interference with the patient [2]. This increased comfort aspect for the patient comes with an increased demand on the engineers to cope with the severe artifacts, which unavoidably occur due to the loose or even non-existing mechanical coupling of sensors and patients [3]. Very robust algorithms are needed, which reliably discard distorted intervals or perhaps allow to compensate for them.

Although many research groups developing sensors and systems for unobtrusive vital signs monitoring or algorithms for signal processing exist [4–15], there is no public database of unobtrusive vital signs signals so far. In case of standard clinical monitoring, the PhysioNet database contains large amounts of clinically acquired signals [16]. The database is used by many groups to develop different algorithms and to compare their results with each other. This is only possible as they all use the same data provided by PhysioNet.

In case of unobtrusive vital signs monitoring, such a database does not exist and thus the results of different signal processing approaches are not comparable. Hence, it is aimed to provide the first publicly available unobtrusive vital signs database called “UnoViS” database. It shall stimulate the research of very robust signal processing algorithms like peak detection, heart rate estimation or sensor fusion, in view of severe and frequent motion artifacts, as these methods are a key aspect for the success of unobtrusive, ubiquitous medical monitoring.

In the following, the data origin, the structure of the database, and its usage are described. In its initial state, the database contains capacitive ECG (cECG) signals (some datasets contain several simultaneously recorded channels) with a reference ECG signal with automatic detected peaks and, in some records, additional optical pulse signals acquired through clothes at the subject’s back. One dataset also contains clinical annotations such as e.g. rhythm analysis, or PQ durations from two clinicians. It is planned to add further sensor technologies like BCG, magnetic induction or video based methods.

## Construction and content

### Origin of data

The origin of the data is manifold and currently consists of three application scenarios: measurements of a clinical study [17], recordings while the subject is driving a car [18, 19] and while lying in bed [20]. Additionally, two records show the maximum measurement quality of our latest system if conditions are optimal. Since the measurements were acquired over a long time period of several years, slightly different (improved) measurement systems were used. In all scenarios, the monitored subjects wore their normal clothes.

#### Measurements from a clinical study

**Figure 1:**
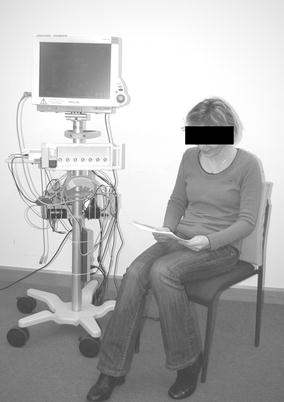
Picture of a patient sitting on the chair equipped with the cushion and the integrated cECG electrodes.

To analyze the reliability and accuracy of non-contact ECG measurements, a clinical study was conducted in the university hospital at RWTH Aachen, Germany. Patients from an anesthesiology premedication and a cardiology day ward were asked to sit in a chair which had a cushion with two integrated capacitive ECG electrodes to derive a bipolar one lead ECG (see Figure 1). The aim was to analyze the sensitivity and specificity for the diagnosis of cardiac arrhythmias utilizing cECG. Further information can be found in the corresponding article [17]. In total, measurements of 92 patients with one annotated 5-s interval are provided. While only undistorted short intervals of 5 s length were used for analysis in the published study, UnoViS provides the complete measurement for each patient. However, the analyzed intervals are marked. The clinical annotations made by two clinicians are based on the analysis of these short intervals.

Each record of the clinical subset contains one cECG and one reference ECG signal (Lead I, obtained with a commercial ICU monitor MP 70, Philips, Netherlands).

#### Measurements while driving a car

**Figure 2:**
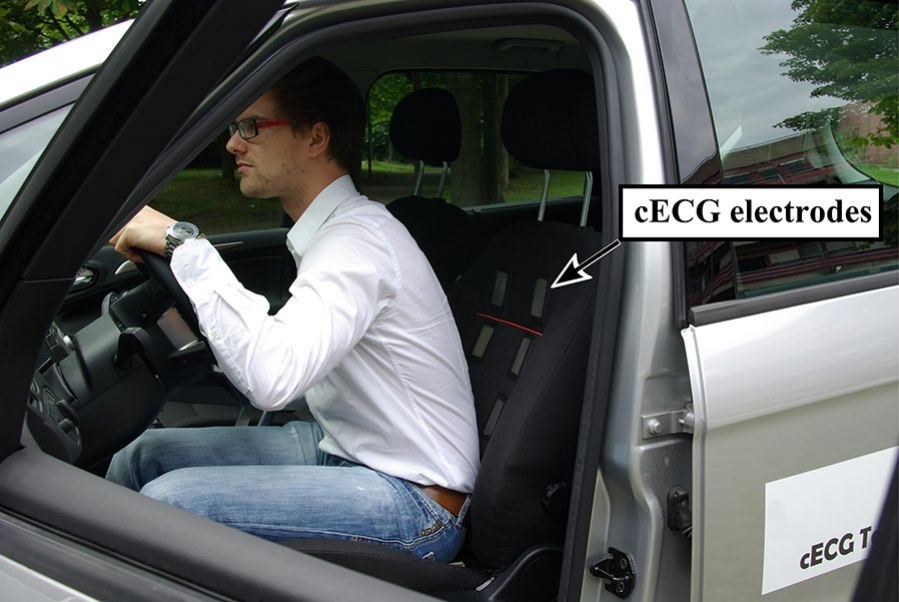
Picture of a driver sitting in the car seat with the integrated electrodes.

In this study, the possibility to measure a cECG of the driver while driving was analyzed. Six volunteers participated as drivers. The system was evaluated while driving in the city, on a highway and on a proving ground in Lommel, Belgium, containing different road types such as highspeed, curvy roads or bad road surfaces. The driver seat contained six electrodes (see Figure 2) from which three cECGs were derived by manually selecting the best three electrodes and taking the corresponding differences:

1 $$\begin{aligned} \text {cECG}_1&= \text {Electrode}_1 - \text {Electrode}_2\end{aligned}$$

2 $$\begin{aligned} \text {cECG}_2&= \text {Electrode}_3 - \text {Electrode}_2\end{aligned}$$

3 $$\begin{aligned} \text {cECG}_3&= \text {Electrode}_3 - \text {Electrode}_1. \end{aligned}$$

To approximate bipolar leads similar to the Einthoven’s triangle, vertically aligned electrodes were not allowed.

Additionally, a reference ECG (Lead I, g.BSamp from g.tec, Austria) was recorded. In contrast to the original corresponding publications [18, 19], UnoViS contains further, previously unpublished records.

#### Measurements while lying in bed

The most recent measurements were performed with an array of 12 electrodes integrated into a mattress on top of a bed and covered with a bed sheet. From those twelve, three were continuously and automatically selected in an online process [21]. This results in a set of three bipolar cECG leads similar to the measurements while driving. Because of the automatic electrode selection, the set of three electrodes may be different between volunteers and can in principle also change during a measurement if the lying position changes a lot. Although this circumstance may complicate the signal analysis, electrode selection is necessary if the sensors are not fixed to the person and the lying position is not known.

**Figure 3:**
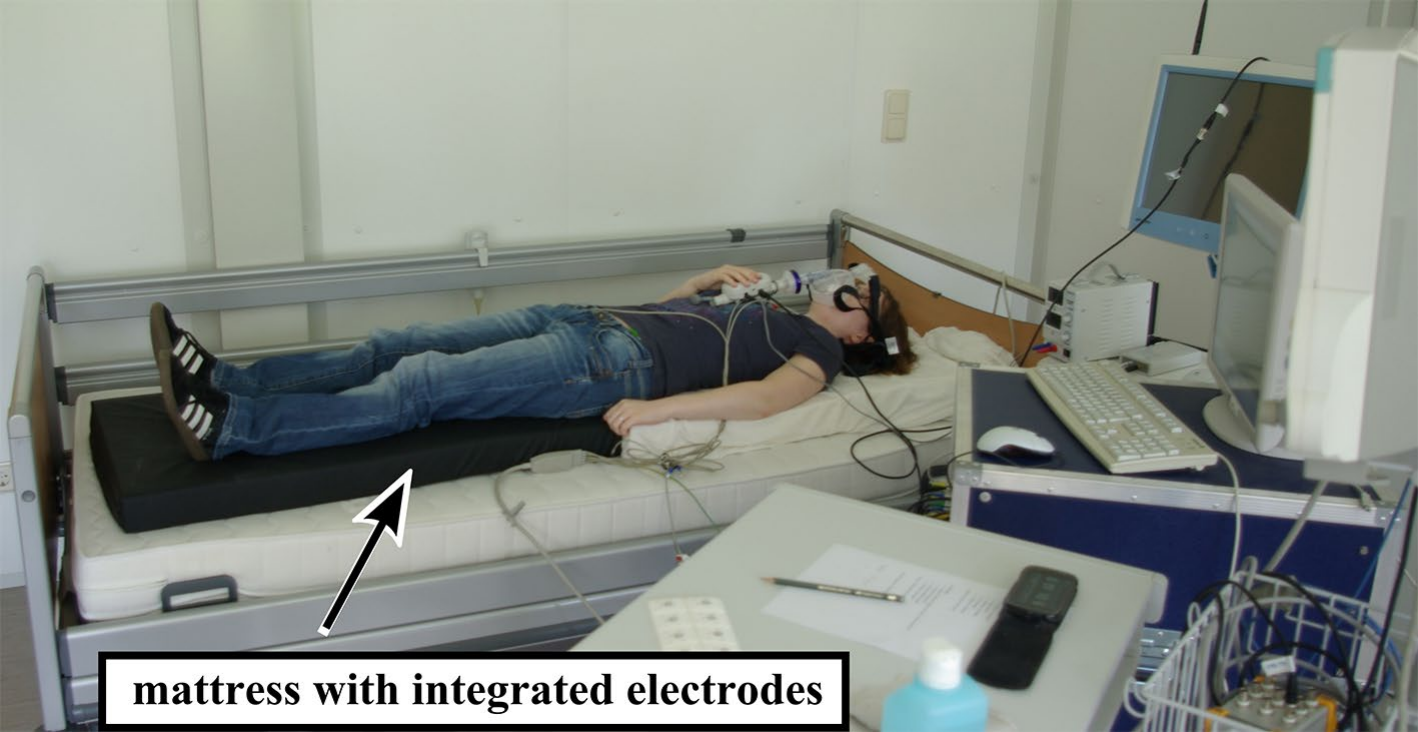
Picture of a person lying in the equipped bed.

Ten volunteers participated and lay down on the sensor-equipped mattress in supine position as it is shown in Figure 3. During the measurements, they performed defined motions following a specific protocol to simulate typical motion artifacts which might occur during sleeping. In ten measurements the volunteers were asked to move every 60 s (denoted with ‘lab, bed, A’ in the dataset). In ten additional measurements they were asked to lie still for 120 s, then move for 60 s and then again lie still for 120 s (denoted with 'lab, bed, B' in the dataset).

As these electrodes have an integrated optical pulse sensor [22], each record contains three cECG, three optical, and one reference ECG signal (Lead I, MP 70, Philips, Netherlands).

#### Measurements under optimal conditions

**Figure 4:**
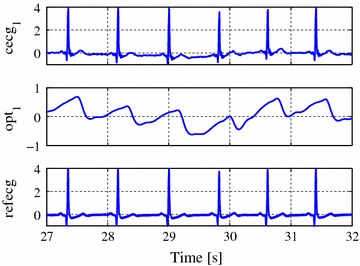
Record of the UnoViS_ opti2013 dataset which shows the signal quality under optimal conditions.

In this case, one subject wearing only a cotton shirt sat on a chair in the lab. Three capacitive electrodes were tightly fixed to the torso with an elastic belt at the standard Einthoven’s triangle position on chest. The two reference ECG electrodes were fixed at the same position (usually named as RA and LA). Hence, the first capacitive ECG lead and the reference ECG measure almost exactly the same signal. Motion was avoided to show that the quality of the cECG is similar to the reference ECG. An example interval is presented in Figure 4. Similar to the measurements before, the record contains three cECG, three optical and one reference ECG signal (Lead I, MP 70, Philips, Netherlands).

### Structure of database

The database consists of records which represent one measurement each. That means, a record contains, for example, one measurement of one patient in case of the clinical study, or one driver driving in a specified scenario in case of the driving tests et cetera. It should be pointed out that several records may exist for the same subject in the driving or lying in a bed scenario. However, these records are labeled with the same unique subject ID but different record IDs.

The database is provided in two formats for easy usage: users who are familiar with PhysioNet can use the data provided in the WFDB file format. Hence, they can use their existing algorithms written in ANSI/ISO C, K&R C, C++, or Fortran for the analysis of data from the UnoViS database [16]. Each record is represented by a header file (e.g. UnoViS_auto2012_1.hea), a data file (e.g. UnoViS_auto2012_1.dat) and an annotation file of the locations of the peaks in the reference ECG signal (e.g. UnoViS_auto2012_1.osearefecg). The information about the measurement scenario, the subject and the quality of the reference ECG is stored in the comment section of the header file.

The structure of each record in case of the MATLAB file is given in Table 1. The presented structure of one record is saved as a struct. Each record consists of several fields such as an unique *id*, the *duration* of the measurement, the measurement scenario *measScenario*, information about the *subject* and several *channels* containing the actual measurement *data* and annotations *ann*.

**Table 1:** Structure of records of the database in MATLAB. The variable* n* just indicates a varying number greater than one and does not mean that* n* is always the same in in all fields or records

Field	Datatype	Occurrence	Content	Example
id	$1 \times n$ char	Always	Unique identifier of each record	UnoViS_auto2012_1
duration	$1 \times 1$ single	Always	Duration of record in seconds	500
measScenario	$1 \times n$ char	Always	Measurement scenario	automotive, city
Subject	$1 \times 1$ struct	Always		
id	$1 \times n$ char	Always	Unique identifier of each subject	p1
clothes	$1 \times n$ char	Optional	Clothes worn	cotton shirt
age	$1 \times 1$ uint8	Optional	Age of subject in years	32
bmi	$1 \times 1$ single	Optional	Body mass index (kg m^−1^)	23.4
sex	$1 \times 1$ char	Optional	Sex	m
Channels	$1 \times n$ struct	Always	Varying number of $n$ $1 \times 1$ structs	
type	$1 \times n$ char	”	Type of channel	cecg
name	$1 \times n$ char	”	Name of channel	cecg_1
fs	$1 \times 1$ single	”	Sampling rate (Hz)	200
data	$n \times 1$ single	”	Raw data (a.u.)	
ann	$1 \times n$ struct	Optional	Varying number of $n$ $1 \times 1$ structs	
type	$n \times 1$ char	”	Type of annotation	peaks
source	$n \times 1$ char	”	Source/origin of annotation	osea
loc	$n \times 1$ uint32	”	Location(s) of annotation(s) (samples)	[25 125 191]

* val* Value of the annotation depends on annotation type. Details are given in Table 2.

The field *ann* is an array of structures within each channel. It contains the *type* (e.g. peaks or rhythm), which may be event based (e.g. in case of the type peaks) or interval based (e.g., in case of the clinical study, the clinicians analyzed an interval of 5 s). It further contains the *source* (e.g. manual by medical experts or automatic by the open source ECG detector OSEA [23]) and the location *loc* of the annotation in samples. The value of the annotation(s) depends on the annotation type and is further elucidated in Table 2. If the annotation refers to an interval, as for example in the clinical study, the field *loc* contains the sample numbers of the analyzed interval. If the annotation is event based, *loc* refers to the location of each event; e.g. of each detected R-peak. Table 2 also shows all annotations defined so far. All ECG signals (capacitive as well as reference) in all datasets contain detected peaks from OSEA and by a medical expert. This allows to compare peak detection algorithms against a gold standard, either based only on the capacitive signals or based on the reference ECG.

**Table 2:** Available annotations

Dataset	Type	Meaning	Source	Value	Datatype
All	peaks	Detected peaks	osea	OSEA Typecode	$1 \times 1$ int8
All	peaks	Detected peaks	manual	OSEA Typecode	$1 \times 1$ int8
UnoViS_clin2009	rhythm	Atrial fibrillation present?	manual	1/0/NaN	$1 \times 1$ logical
UnoViS_clin2009	extrasys	Extrasystole present?	manual	1/0/NaN	$1 \times 1$ logical
UnoViS_clin2009	bbb	Bundle branch block?	manual	1/0/NaN	$1 \times 1$ logical
UnoViS_clin2009	hr	Heart rate	manual	[mean, relative difference]	$2 \times 1$ single
UnoViS_clin2009	pq	PQ time	manual	[mean, relative difference]	$2 \times 1$ single
UnoViS_clin2009	qrs	QRS time	manual	[mean, relative difference]	$2 \times 1$ single
UnoViS_clin2009	qt	QT time	manual	[mean, relative difference]	$2 \times 1$ single

In case of the measurements from the clinical study, this dataset contains the following annotations for the cECG as well as for the reference ECG:

- *rhythm*: is atrial fibrillation present?
- *extrasys*: are extrasystoles present?
- *bbb*: is a bundle branch block present?

All these annotations specify only if it occurs somewhere in the 5-s interval. If the two clinicians could not clearly identify a parameter or if their results differed on the given 5-s interval, it is denoted with *NaN*. Furthermore, the heart rate and different time durations such as PQ, QRS, and QT duration are given. Here, the mean value of the two clinicians’ results and the relative difference are given. Again, if one parameter, e.g. the PQ duration, could not be clearly estimated on the given 5-s interval, it is denoted with *NaN*.

### Statistics of database

In its current state, the database contains 145 records with a total duration of 16.2 h of data. As several channels exist in some records, the amount of raw data is even greater (46.5 h for capacitive ECG signals, 5.4 h for optical signals and 16.2 h for the reference ECG signals, see also Table 3. "#Rec." means number of records, "Dur. refECG" means total duration of reference ECG). In the following, all values are given in mean ± two standard deviation.

**Table 3:** Summary of initial content of database

Dataset	Signaltypes	#Rec.	Dur. refECG
UnoViS_clin2009	1 Capacitive ECG	92	55 min
	1 Reference ECG		
UnoViS_auto2012	3 Capacitive ECGs	31	13.4 h
	1 Reference ECG		
UnoViS_bed2013	3 Capacitive ECGs	20	1.7 h
	3 Optical pulse		
	1 Reference ECG		
UnoViS_opti2013	3 Capacitive ECGs	2	6.3 min
	3 Optical pulse		
	1 Reference ECG		

#### Clinical study

In case of the clinical study (*measScenario: ‘clinicalStudy2009’*), 92 subjects are included with a total duration of 55 min. The age of the patients is 64.3 ± 21.9 years and the BMI is 27.7 ± 8,9 kg m^−1^. The gender was not recorded.

#### Driving a car

The measurements while driving a car are composed of 31 records of 6 different subjects resulting in 13.4 h of data. They have an age of 39.8 ± 26.2 years and a BMI of 27.0 ± 11,6 kg m^−1^. All volunteers are male.

The distribution of duration per specific scenario is as follows:

- *‘automotive, city*': 2.0 h
- *‘automotive, highway*': 8.8 h
- *‘automotive, proving ground*': 2.5 h

here, ‘automotive, *' are the identifiers found in the field *measScenario* of the respective record.

#### Lying in bed

The measurements where a subject is lying in bed (*measScenario: ‘lab, bed, A’, ‘lab, bed, B’*) consist of 20 records of 10 different subjects resulting in 1.7 h of data. The volunteers have and age of 27.8 ± 4.3 years and a BMI of 24.2 ± 8.3 kg m^−1^, while 30% are female.

#### Optimal conditions

The measurement under optimal conditions consists of two records (*measScenario: ‘lab, chair, optimal conditions’*) of one subject and a total duration of 6.3 min. The male subject’s age is 28 and he has a BMI of 22.2 kg m^−1^.

## Utility

**Figure 5:**
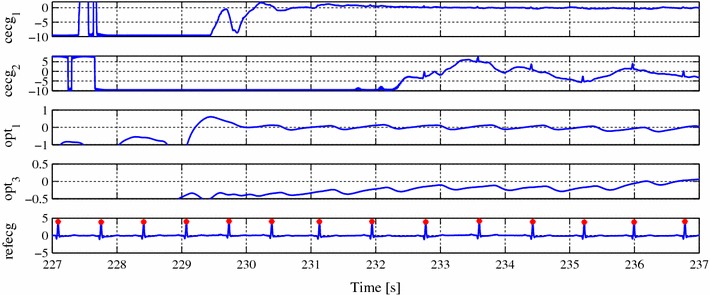
Example plot of a multichannel record (first record from dataset ”UnoViS_bed2013”) with artifacts.

Many signal processing challenges exist in the realm of unobtrusive monitoring. One important aspect is the peak detection, i.e. the precise location of individual heart beats. Subsequently, parameters like heart rate (HR) or heart rate variability (HRV) can be calculated. Nonetheless, it is also possible to estimate the heart rate with indirect methods [24–26]. However, especially in unobtrusive monitoring without fixed electrode-patient-interfaces, severe artifacts are prevalent and large intervals might be corrupted to a degree that the extraction of useful information is impossible. It is hence equally important to detect these corrupted intervals and exclude them from further analysis. In case of several simultaneously recorded redundant signals, it is furthermore possible to perform signal fusion to increase the temporal coverage of valid information and simultaneously decrease the error, e.g. in HR estimation as we have shown in [27].

All these mentioned scenarios (and probably even more) are possible with UnoViS. An example for the severe motion artifacts and the opportunities of a multichannel unobtrusive monitoring is shown in Figure 5. While the channel cecg$_1$ has a very low amplitude of the R-peaks even after recovering from a severe artifact, channel cecg$_2$ shows larger amplitudes of the R-peaks. Hence, it would be beneficial to use this channel for parameter extraction. Furthermore, the optical channels opt$_1$ and opt$_3$ recover even faster and would thus allow to increase the time in which a reliable heart rate could be estimated.

### Provided functions

For the purpose of easy usage and fast development of own algorithms, several MATLAB functions are provided with the database. In case of the WFDB files, it is possible to start immediately out of the box with own existing algorithms, as data is stored in a standardized file format. However, a plot function for MATLAB, which loads a WFDB file and plots all available channels and information, is attached as an example.

For the database stored in a MATLAB mat-file, basic working examples for peak and artifact detection as well as result analysis are provided. The usage of those functions and procedures are demonstrated in the script *EXAMPLE_USAGE.m*. They can be used with a single record or, for increased statistical power, with the whole database. These functions allow researchers to concentrate on the development of sophisticated algorithms instead of having to build the needed infrastructure and the evaluation methods.

#### Detection of peaks

The detection of heart beats in unobtrusively measured signals is a challenging task. For demonstration, *findPeaksInRecord* uses the OSEA QRS-detector to detect R-peaks in capacitive ECG signals of a given record. To evaluate its performance, *bxbAmsi* calculates the sensitivity and positive predictive value (PPV) of the detected peaks compared with the given reference peaks. Calculations are based on the two standards ANSI/AAMI EC38:1998, the American National Standard for Ambulatory ECGs, and ANSI/AAMI EC57:1998, the American National Standard for Testing and Reporting Performance Results of Cardiac Rhythm and ST Segment Measurement Algorithms.

It is also possible to perform an artifact detection after peak detection to classify each detected peak using a quality index. This is demonstrated with a simple algorithm called *calcQualityIndex*, which discards every beat that would result in a heart rate above 120 BPM and below 30 BPM. In case of a heavily distorted signal resulting in many additional (falsely) detected peaks, this would decrease the sensitivity but increase the positive predictive value. This very simple approach is of course not sufficient for a real application but can serve as a minimal working example for future developments.

#### Error and temporal coverage of heart rate estimation

As already stated, beat-to-beat heart rates can be estimated based on detected peaks or by other, *indirect* methods [24–26]. In any case, algorithms need to be evaluated by two parameters: error and temporal coverage. It is obviously important that the error compared to a reference heart rate should be as low as possible. However, a high temporal coverage is important as well - one could exclude all “challenging” data segments and get very low errors, but at the same time almost no information. The function *calcHrErrorTimeCoverage* calculates these two parameters. The error $e_{\text {HR}}$ is calculated based on an upsampled and interpolated (in a staircase way) heart rate $\text {HR}^{\text {up}}_{\text {test}}$ of the test signal instead of a beat-to-beat heart rate. The error, i.e., the difference between the test and the reference heart rate, is calculated at the peak positions of the beat-to-beat reference heart rate $t_\text {ref,peak}$.^a^

4 $$\begin{aligned} e_{\text {HR}} = \text {HR}^{\text {up}}_{\text {test}}(t_\text {ref,peak}) - {\text {HR}}^{\text {b2b}}_{\text {ref}} \end{aligned}$$

The temporal coverage is calculated as the ratio of time in which information is available in both the upsampled reference and test heart rate, and the total time in which information is available in the upsampled reference heart rate. Since samples with no information contain NaN, the calculation of the temporal coverage can be expressed as

5 $$\begin{aligned} \text {coverage} = \frac{t(\text {HR}^{\text {up}}_{\text {test}} \ne \text {NaN} \ \& \ \text {HR}^{\text {up}}_{\text {ref}} \ne \text {NaN})}{t(\text {HR}^{\text {up}}_{\text {ref}} \ne \text {NaN})} \end{aligned}$$

These definitions help to compare artifact detection algorithms, which discard corrupted intervals. If no artifact detection is utilized, the error $e_{\text {HR}}$ may be large and the temporal coverage is 100%. If corrupted intervals are discarded, the error reduces but also the coverage in which information is available (without differentiation if available information is correct or not). A good algorithm will result in a minimal error and a maximal temporal coverage.

For presentation of the results *boxPlotErrorsCoverages* plots a box plot of the errors and the temporal coverage. Again, it is also possible to use quality indexes and *boxPlotErrorsCoverages* will show their impact in a direct comparison to the unclassified peaks.

**Figure 6:**
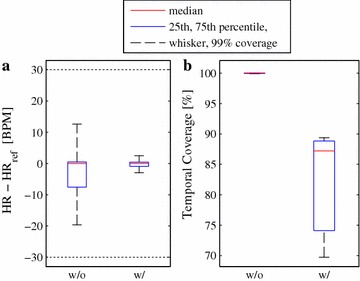
Example of a* box plot* to show the effect of an artifact detection algorithm.* w/o* Without artifact detection,* w/* with artifact detection.** a** Error in heart rate,** b** temporal coverage.

As an example, one record from the database "UnoViS_auto2012”, containing three cECG channels, is extracted. A simple artifact detection method is used and the results are presented as a box plot in Figure 6. The figure shows (a) the error of the heart rate detection and (b) the temporal coverage. The red line denotes the median, the blue box contains 50% of all data points, and the whiskers show the distribution of 99% of all data points. In case of the heart rate error, the errors above 30 BPM and below −30 BPM are moved to these limits, to remain legible.

It is clearly visible that, in case of no artifact detection, the errors in heart rate estimation are broadly distributed. 50% have an error between −8 and 1 BPM, however, 99% are between −20 and 12 BPM (keeping in mind that even larger errors are limited to ±30 BPM) and are hence not acceptable. The temporal coverage is at 100%. If the simple artifact detection is applied, the heart rate estimation improves significantly as most errors are now within an interval of ±5 BPM. However, as it is an overly simple artifact detection method, many large errors remain, which are not visible here due to the set limits. The temporal coverage becomes, as expected, lower and lies between 70 and 90%.

## Discussion

The purpose of the database is not to set a new standard but rather to provide a database for heavily distorted unobtrusive vital signs measurements. Different measurement devices by different research groups worldwide will of course result in slightly different measurements. However, they all have to conquer severe motion artifacts. Although these measurement devices are not commercially available yet, they are gaining more and more attention in research as well as in industry. Hence, to allow all researchers to develop very robust algorithms, this database closes an important gap as it is the first of its kind. There is no need to own such a device and the results can be compared with others.

## Conclusions

This paper presents the first publicly available database of unobtrusive vital signs measurements. Beside the structure and the initial content of the database itself, supplementary MATLAB functions are described. They allow prospective users an easy usage of the database and fast developing and testing of their own algorithms. Especially for peak detection, heart rate estimation and artifact classification, working example algorithms and evaluation functions are provided. This helps to avoid their time consuming yet required implementation for prospective database users. In the future, this database will be extended with more cECG measurements and other unobtrusive vital signs data acquired by various technologies such as, for example, BCG, video or magnetic impedance measurements.

## Availability and requirements

The database is located at http://www.medit.hia.rwth-aachen.de/UnoViS and can be downloaded for free. However, we kindly ask to cite this publication if the database and/or its supplementary functions are used. The data itself is available as a MATLAB mat-file or in several files per record in accordance to the PhysioNet WFDB (WaveForm DataBase) file format [16].

## Endnotes

^a^It should be noted, that the positions are slightly shifted to avoid wrong large differences at the heart rate transitions.

## References

[1] Scalise L, Millis RM (2012). Non contact heart monitoring. Advances in electrocardiograms—methods and analysis.

[2] Zheng Y-L, Ding X-R, Poon CCY, Lo BPL, Zhang H, Zhou X-L (2014). Unobtrusive sensing and wearable devices for health informatics. IEEE Trans Bio-Med Eng.

[3] Sweeney KT, Ward TE, McLoone SF (2012). Artifact removal in physiological signals-practices and possibilities. IEEE Trans Inf Technol Biomed.

[4] Baek HJ, Chung GS, Kim KK, Park KS (2012). A smart health monitoring chair for nonintrusive measurement of biological signals. IEEE Trans Inf Technol Biomed.

[5] Spinelli E, Haberman M, García P, Guerrero F (2012). A capacitive electrode with fast recovery feature. Physiol Meas.

[6] Chi YM, Maier C, Cauwenberghs G (2011). Ultra-high input impedance, low noise integrated amplifier for noncontact biopotential sensing. IEEE J Emerg Sel Top Circ Syst.

[7] Serteyn A, Vullings R, Meftah M, Bergmans JWM (2015). Motion artifacts in capacitive ECG measurements: reducing the combined effect of DC voltages and capacitance changes using an injection signal. Biomed Eng IEEE Trans.

[8] Heuer S, Martinez DR, Fuhrhop S, Ottenbacher J (2009) Motion artefact correction for capacitive ECG measurement. In: IEEE Biomedical Circuits and Systems Conference (BioCAS), pp 113–116

[9] Kato T, Ueno A, Kataoka S, Hoshino H, Ishiyama Y (2006) An application of capacitive electrode for detecting electrocardiogram of neonates and infants. In: 28th Annual International Conference of the IEEE Engineering in Medicine and Biology Society (EMBS). IEEE, New York 10.1109/IEMBS.2006.260362 17946008

[10] Peng G, Bocko MF (2013). Non-contact ECG sensing employing gradiometer electrodes. Biomed Eng IEEE Trans.

[11] Kranjec J, Begus S, Drnovsek J, Gersak G (2014). Novel methods for noncontact heart rate measurement: a feasibility study. IEEE Trans Instrum Meas.

[12] Ichapurapu R, Jain S, John G, Monday T, Lie D, Banister R, et al. (2009) A 2.4 GHz non-contact biosensor system for continuous vital-signs monitoring. In: IEEE 10th Annual Wireless and Microwave Technology Conference. IEEE, New York, pp 1–3.

[13] Terrence J, Shinar Z, Klap T, Brown H (2009) Contact-free under-the-mattress monitoring for early recognition of end-of-life in Med/Surg units. In: 8th International Conference on Rapid Response Systems and Medical Emergency Teams

[14] Mack DC, Patrie JT, Suratt PM, Felder RA, Alwan MA (2009). Development and preliminary validation of heart rate and breathing rate detection using a passive, ballistocardiography-based sleep monitoring system. IEEE Trans Inform Technol Biomed.

[15] Clippingdale AJ, Prance RJ, Clark TD, Watkins C (1994). Ultrahigh impedance capacitively coupled heart imaging array. Rev Sci Instrum.

[16] Goldberger AL, Amaral LAN, Glass L, Hausdorff JM, Ivanov PC, Mark RG (2000). PhysioBank, PhysioToolkit, and PhysioNet: components of a new research resource for complex physiologic signals. Circulation.

[17] Czaplik M, Eilebrecht B, Walocha R, Walter M, Schauerte P, Leonhardt S (2012). The reliability and accuracy of a noncontact electrocardiograph system for screening purposes. Anesth Analg.

[18] Eilebrecht B, Wartzek T, Lem J, Vogt R, Leonhardt S (2011). Capacitive electrocardiogram measurement system in the driver seat. Automobiltechnische Zeitschrift ATZ.

[19] Wartzek T, Eilebrecht B, Lem J, Lindner H-J, Leonhardt S, Walter M (2011). ECG on the road: robust and unobtrusive estimation of heart rate. IEEE Trans Biomed Eng.

[20] Wartzek T, Brüser C, Schlebusch T, Brendle C, Santos S, Kerekes A et al (2013) Modeling of motion artifacts in contactless heart rate measurements. In: Computing in Cardiology (CinC 2013)

[21] Wartzek T, Weber H, Walter M, Eilebrecht B, Leonhardt S (2012) Automatic electrode selection in unobtrusive capacitive ECG measurements. In: 25th International Symposium on Computer-Based Medical Systems (CBMS 2012)

[22] Wartzek T, Elfring R, Jansen A, Eilebrecht B, Walter M, Leonhardt S (2011) On the way to a cable free operating theater: an operating table with integrated multimodal monitoring. In: Computing in Cardiology (CinC 2011)

[23] Hamilton P (2002) Open source ECG analysis. In: Computers in cardiology, 22–25 Sept 2002. IEEE, pp 101–104. doi:10.1109/CIC.2002.1166717

[24] Brüser C, Winter S, Leonhardt S (2013). Robust inter-beat interval estimation in cardiac vibration signals. Physiol Meas.

[25] Kortelainen JM, Virkkala J (2007) FFT averaging of multichannel BCG signals from bed mattress sensor to improve estimation of heart beat interval. In: Engineering in Medicine and Biology Society, 29th Annual International Conference of the IEEE, pp 6685–6688 10.1109/IEMBS.2007.4353894 18003560

[26] Kortelainen JM, van Gils M, Parkka J (2012) Multichannel bed pressure sensor for sleep monitoring. In: Computing in Cardiology (CinC), pp 313–316

[27] Wartzek T, Bruser C, Walter M, Leonhardt S (2014). Robust sensor fusion of unobtrusively measured heart rate. IEEE J Biomed Health Inform.

